# Cytogenetic analysis of the pathology of frozen shoulder

**DOI:** 10.4103/0973-6042.76966

**Published:** 2010

**Authors:** Benjamin Kabbabe, Satish Ramkumar, Martin Richardson

**Affiliations:** 1 The University of Melbourne, Parkville, Australia; 2 The University of Melbourne, Parkville; The Royal Melbourne Hospital, Grattan St Parkville, Australia; 3 The Epworth Hospital, 89 Bridge Rd Richmond, Victoria, Australia

**Keywords:** Cytokines, diabetes mellitus, frozen shoulder, inflammation, matrix metalloproteinase

## Abstract

**Background:**

Frozen shoulder (FS) is a debilitating musculoskeletal condition with an uncertain etiology and pathogenic mechanism. The aim of this study was to investigate the hypothesis that an alteration in the level of cytokines may disrupt the normal inflammatory and tissue healing process in the shoulder, leading to the development of FS.

**Materials and Methods:**

A prospective case–control study was undertaken, analyzing patients undergoing arthroscopic treatment of FS and control patients being treated for subacromial bursitis. Synovial biopsies were taken from all subjects. Synovial RNA levels were analyzed using quantitative polymerase chain reaction (qPCR)

**Results:**

Thirteen patients with FS were recruited, four of whom were diagnosed with diabetes mellitus, along with 10 control patients. Cytogenetic analysis using qPCR revealed both fibrogenic cytokine matrix metalloproteinase 3 (MMP 3) (1.98×10 ^5^ vs. 755.0, *P*=0.068) and inflammatory cytokine interleukin 6 (IL 6) (1679.2 vs. 372.8, *P*=0.062) to be elevated in FS cases as compared to controls. Comparison between diabetic and non-diabetic patients revealed a decrease in the level of expression of inflammatory cytokine, monocyte colony stimulating factor (M-CSF) (12,496 vs. 305.1, *P*=0.04) in diabetic FS patients.

**Conclusions:**

The results demonstrate that levels of inflammatory and fibrogenic cytokines are elevated in the synovium of patients with FS compared with controls. This indicates that altered levels of inflammatory cytokines may be associated with the pathogenesis of inflammation evolving into fibrosis, which is the characteristic feature of FS. We have also shown the opposite to be the case in patients with diabetic FS.

## INTRODUCTION

Frozen shoulder (FS) describes a condition of shoulder pain and stiffness of unknown etiology. It is a slowly progressive disease, beginning with shoulder pain that is worse at night, due to an underlying inflammatory process affecting the joint capsule and shoulder ligaments.[1] The range of movement of the shoulder decreases over a period of months until it becomes non-functional.

Secondary FS is diagnosed when there is an underlying pathology or disorder that has been previously associated with an increase in the prevalence of FS, compared with those without the condition. Diabetes mellitus (DM) is the most common association of secondary FS.[2] The incidence of FS in diabetic patients is 10–36%, compared with 2–10% in non-diabetic patients.[3–7] The condition is often more severe, more painful, and less responsive to conservative treatment in diabetic patients.[3,6,8–10]

This study was designed to primarily to investigate the pathogenic mechanisms that underlie the development and progression of FS. This was largely done to better understand and characterize the potential mechanisms suggested by previous authors. In particular, we wanted to define the role of various cytokines and growth factors in orchestrating the development of FS.

Secondarily, the study sought to investigate whether there was a difference in the pathology that affects those suffering from primary FS and secondary FS, which might explain the wide spectrum of morbidity seen in FS, and may lead to more targeted treatment modalities.

## MATERIALS AND METHODS

A prospective case–control study was conducted between August 2007 and August 2008 at the Epworth Hospitals, Melbourne. The study was conducted after obtaining ethics approval from the Epworth Hospital Ethics Committee (Epworth study number 28304). The purpose of the study was to investigate the pathogenesis of FS and the changes to the synovium in FS patients. All patients were selected from the Epworth Hospital in either Richmond or Box Hill as patients of the same orthopedic surgeon (MR).

Four sections of synovial tissue, measuring approximately 1 cm×1 cm, were taken intraoperatively from four different locations within the joint and placed in a specimen container containing normal saline. RNA analysis was performed by staff at the Hamilton Lab, Department of Medicine (Royal Melbourne Hospital). The staff did not have access to the patient’s medical history and were not aware of their surgical findings. A laboratory technician, blind to the clinical data and surgical findings, performed a quantitative polymerase chain reaction (qPCR) analysis. The findings were expressed as a numerical value, relative to the level of expression of 18s mRNA, a universal housekeeper gene, and were analyzed using SPSS statistical software.[11]

Fibrogenic cytokines analyzed included matrix metalloproteinase 13 (MMP 13), matrix metalloproteinase 1 (MMP 1), matrix metalloproteinase 3 (MMP 3), A disintegrin and metalloproteinase with thrombospondin motifs 4 (ADAMTS 4), and A disintegrin and metalloproteinase with thrombospondin motifs 5 (ADAMTS 5).

Inflammatory cytokines analyzed included tumour necrosis factor α (TNF α), interleukin 1b (IL 1b), interleukin 6 (IL 6), interleukin 8 (IL 8) and growth factors granulocyte-macrophage colony stimulating factor (GM-CSF) and macrophage colony stimulating factor (M-CSF).

## RESULTS

The study included 13 cases clinically diagnosed with FS by the treating orthopedic surgeon, along with 10 control patients diagnosed with rotator cuff tears. There were four diabetic FS cases, all with type 2 diabetes, self-reported as being previously diagnosed by a medical professional.

Arthroscopic visualization of the glenohumeral joint revealed evidence of inflammatory changes and synovitis in all FS cases. There was a build-up of scar tissue and adhesions within the capsule, and the capsule was found to be inflamed, thickened and demonstrate more proliferative tissue, in comparison to controls.

Analysis of inflammatory cytokines and growth factors revealed a high level of similarity between FS samples and controls. Two cytokines of note are IL 6 and IL 8, both of which had higher levels of expression in FS samples. Results for the other cytokines tested had similar levels of expression between the two groups, and are difficult to interpret [Table 1]

**Table 1:** Comparison of inflammatory cytokines of frozen shoulder subjects and controls

Inflammatory cytokine		Frozen shoulder	Control	*P*
IL 1b	Median(IQR)	154.0(20.4−1079.3)	12.7(3.7−188.1)	0.25
IL6	Median(IQR)	1679.2(569.5−5061.2)	372.8(125.1−1304.5)	0.06
IL8	Median(IQR)	362.0(42.1−1018.0)	58.6(31.5−90.9)	0.08
TNF α	Median(IQR)	484.8(254.2−1176.3)	152.6(77.0−12.9)	0.34
M-CSF	Median(IQR)	8726.1(5878.1−12,712.2)	3773.8(2567.6−13,085.9)	0.63
GM-CSF	Median(IQR)	73.1(47.3−181.8)	32.7(11.2−1034.3)	0.39

IL = Interleukin; IQR =Interquartile range; TNF α = Tumor necrosis factor alpha; M-CSF = Monocyte colony stimulating factor; GM-CSF = Granulocyte-macrophage colony stimulating factor

Statistical analysis of the cytokines involved in fibrosis revealed the levels of MMP 3 and ADAMTS 4 to be elevated in the FS group compared to controls. Two of the nine FS patients had zero expression of MMP 13, as did three of the eight control patients. It was also noted that two of nine FS patients had zero expression of MMP 1, compared with one of eight control samples. No other results showed zero expression of any other cytokine in either group[Table 2].

**Table 2:** Comparison of fibrogenic cytokines of frozen shoulder subjects and controls

Fibrogenic cytokine		Frozen shoulder	Control	*P*
MMP 1	Median(IQR)	9369.4(13.9−11,647.9)	235.4(43.9−2006.0)	0.39
MMP 3	Median(IQR)	19,792.1(5286.5−26,2883.6)	755.0(444.6−8700.6)	0.07
MMP 13	Median(IQR)	29.7(4.5−119.3)	1.6(018.9)	0.12
ADAMTS 4	Median(IQR)	702.45(531.4−2129.9)	371.0(200.4−866.2)	0.08
ADAMTS 5	Median(IQR)	333.0(206.8−1079.3)	1090.1(364.1−5118.0)	0.50

MMP = Matrix metalloproteinase; IQR = Interquartile range; ADAMTS = A disintegrin and metalloproteinase with thrombospondin motifs

The FS sample group was then subdivided into diabetic and non-diabetic cases. Statistical analysis of the findings from these two groups revealed a high degree of similarity. Of note was the significant increase in the level of M-CSF expression in the non-diabetic FS group, compared with the diabetic group [Table 3].

**Table 3:** Comparison of inflammatory cytokines of diabetic and non-diabetic frozen shoulder subjects

Inflammatory cytokine		Diabetic FS	Non−diabetic FS	*P*
IL 1b	Median(IQR)	14.1(9.9−142.7)	216.0(65.7−884.6)	0.43
IL 6	Median(IQR)	563.8(283.6−2286.1)	1786.0(1079.6−12,044.0)	0.21
IL 8	Median(IQ)R	603.7(307.9899.4)	382.4(232.71023.5)	0.21
TNF α	Median(IQR)	350.3(105.3−807.1)	484.8(395.3−1176.3)	0.51
M-CSF	Median(IQR)	305.1(177.2−679.4)	12,496.0(9615.8−14,320.2)	0.04
GM-CSF	Median(IQR)	1838.8(1498.3−5134.0)	60.3(32.3−103.2)	0.30

IL = Interleukin; IQR = Interquartile range; TNF α = Tumor necrosis factor alpha; M-CSF = Monocyte colony stimulating factor; GM-CSF = Granulocyte-macrophage colony stimulating factor

Comparison of the level of cytokines involved in fibrosis between diabetic and non-diabetic FS cases revealed a significant increase in the level of expression of the metalloproteinases MMP 1 and ADAMTS 5 in non-diabetic FS patients [Table 4].

**Table 4:** Comparison of fibrogenic cytokines of diabetic and non-diabetic frozen shoulder subjects

Fibrogenic cytokine		Diabetic FS	Non-diabetic FS	*P*
MMP1	Median(IQR)	14.0(7.0−4586.2)	10,830.8(9550.7−15,400.3)	0.09
MMP 3	Median(IQR)	5286.5(3944.2−13,221.9)	141,337.8(12,384.2−355,511.5)	0.38
MMP 13	Median(IQR)	4.5(2.2−17.1)	95.6(34.6−579.8)	0.12
ADAMTS 4	Median(IQR)	531.4(385.9−1361.1)	1040.7(648.5−1942.2)	0.44
ADAMTS 5	Median(IQR)	196.4(129.8−239.1)	957.1(458.4−2384.0)	0.04

MMP = Matrix metalloproteinase; IQR = Interquartile range; ADAMTS = A disintegrin and metalloproteinase with thrombospondin motifs

## DISCUSSION

The results of this study have revealed several new elements of this enigmatic condition, as well as raised new questions about the basic pathophysiological mechanism of FS. The cellular signaling pathways involved in FS have previously been investigated to ascertain whether they are pathogenic. It has been suggested that cytokines may act as a persistent fibrogenic stimulus, causing capsular fibrosis and the development of FS.[12] MMPs and the inhibitors of matrix metalloproteinases (TIMPs) regulate the remodeling of the extracellular matrix that the fibroblasts produce. High levels of MMP have been found in FS patients; however, even higher levels of TIMP have been found in the same tissue.[12] These findings are consistent with a trial conducted by Hutchison[13] in which a TIMP analogue (Marimastat) was given as an anti-cancer treatment to patients suffering from gastric carcinoma. Of the 12 that took the treatment, six had developed bilateral FS within 4 months and three had developed a palmar contracture. These findings suggest that FS is an inflammatory condition that progresses to a fibrotic capsular contracture due to an imbalance in the cytokines and growth factors that regulate the normal healing response.[14]

The use of qPCR to assess the level of mRNA expression of cytokines and growth factors in shoulder synovial cells is novel in the field of FS research. Most of the FS cases had a prolonged history of the disease and were being treated late in their presentation. This meant that the acute inflammatory phase of FS should have been subsiding. However, we found the inflammatory cytokines IL 6 and IL 8, both of which are downstream molecules involved in the acute inflammatory reaction, to be elevated. This finding tends to suggest that there is still some degree of residual inflammation that carries on in late stage FS. These findings are similar to those of Bunker *et al*.,[12] who found an increase in the expression of interleukins in FS patients. Furthermore, this level of inflammation is above that seen in a mechanical injury, as represented by the control subjects. A potential explanation for this increase is that the pathogenesis of FS involves an up-regulation of inflammatory cytokines, either as part of a chronic inflammatory process or due to an underlying physiological abnormality. This would explain why some patients heal normally in response to injurious stimuli, while others go on to develop FS.

Given the natural history of FS and the timing of the surgery, it was expected that many of the FS patients would be entering the fibrotic stage of the disease. We found that the levels of the MMP 3 and ADAMTS 4 were both elevated in FS patients. This elevation supports the initial hypothesis that these factors are involved in the evolution of FS from an inflammatory condition to a fibrotic one. However, these results are in contradiction to findings by previous authors[12 13] who suggested that it is an elevation in TIMP and a subsequent decrease in the metalloproteinases that results in fibrosis. Our findings suggest that this model is potentially flawed, as it does not take into account the evolution of the condition from an inflammatory to a fibrotic one. We also found several FS and control samples had zero expression of MMP 1 and MMP 3, which was unique to these cytokines in our analysis. Interestingly, if the frequency measure used in the study by Bunker *et al*.[12] had been used here, we would have also found a reduced expression of MMPs. An explanation for this may be that after the expression of MMPs has spiked as part of the up-regulation of the normal healing and regenerative process, MMP expression is completely inhibited due to excessive TIMP inhibition as part of a negative feedback cycle. Therefore, the triggering factor of FS could be an excessive inflammatory response, with resulting elevations in factors involved in the healing cascade, such as metalloproteinases and growth factors. This could in turn result in an equally excessive inhibition of this process via a negative feedback loop or as a result of the same common pathology. The FS patient is therefore unable to resolve the excessive inflammatory reaction, leading to a protracted, fibrotic shoulder.

A secondary question of this study was whether the pathogenic process differed between diabetic and non-diabetic FS cases. Significant differences in the pathogenesis of diabetic FS have not been found previously.[12 15] Cytogenetic analysis revealed inflammatory cytokines to be similar between the two groups, and this goes some way in answering the initial question posed. However, the results also demonstrate that there is a significant decrease in the amount of the inflammatory growth factor M-CSF and the ADAMTS 5 cartilage-cleaving enzyme in diabetic FS cases. The cause of this decrease is not known, and the implications of this in diabetic cases remain speculative. It may be that monocytes are under-stimulated in diabetic cases, which would result in a slowed, delayed, or aberrant inflammatory response. If this were the case, it might be a cause for the evolution of a normal inflammatory reaction into a chronic fibrotic response. It is known that diabetics have poor wound healing and are susceptible to microangiopathy, and these factors may combine with low expression of M-CSF to result in a poor response to an initially innocuous inflammatory trigger. In turn, this could produce an FS condition that is more severe, more painful, and less responsive to conservative management, which focuses on suppression of inflammation.

This study suffers from several limitations. Most notably, the number of patients recruited was less than hoped for, and therefore, the tests for statistical significance for values obtained were often above the pre-designated level of significance where the null hypothesis can be ruled out. Larger patient numbers would have also resulted in a more accurate representation of the population being tested. Another issue was that all patients were sourced from the same private hospital. It is also known that private hospital patients tend to be more healthy than those being treated in public hospitals and are generally treated much sooner than those in the public system.

## CONCLUSION

FS has always been a complicated and poorly understood condition. We have shown that there are quantifiable differences in the pathological appearance of FS. These differences can be observed clinically, arthroscopically, and are also found in the molecular expression of cells of an affected shoulder. These differences demonstrate that the pathogenesis of FS is caused by the evolution of a controlled inflammatory response to an unknown injurious stimulus into an aberrant fibrotic process. Several cytokine messengers involved in the normal healing process were analyzed. The results demonstrate that levels of expression of several cytokines were elevated. This elevation may be the cause of the alteration in the normal healing process that leads to the development of FS. Hopefully, with a greater level of understanding, we can offer patients effective levels of therapy that help to alleviate their pain and suffering, and one day offer a cure to this debilitating condition.
